# Asthma Diagnosed after 11 September 2001 among Rescue and Recovery Workers: Findings from the World Trade Center Health Registry

**DOI:** 10.1289/ehp.10248

**Published:** 2007-08-27

**Authors:** Katherine Wheeler, Wendy McKelvey, Lorna Thorpe, Megan Perrin, James Cone, Daniel Kass, Mark Farfel, Pauline Thomas, Robert Brackbill

**Affiliations:** 1 New York City Department of Health and Mental Hygiene, New York, New York, USA; 2 New Jersey Medical School, UMDNJ, Newark, New Jersey, USA; 3 Agency for Toxic Substances and Disease Registry, Atlanta, Georgia, USA

**Keywords:** asthma, disaster, masks, respirators, World Trade Center, workers

## Abstract

**Background:**

Studies have consistently documented declines in respiratory health after 11 September 2001 (9/11) among surviving first responders and other World Trade Center (WTC) rescue, recovery, and clean-up workers.

**Objectives:**

The goal of this study was to describe the risk of newly diagnosed asthma among WTC site workers and volunteers and to characterize its association with WTC site exposures.

**Methods:**

We analyzed 2003–2004 interview data from the World Trade Center Health Registry for workers who did not have asthma before 9/11 (*n* = 25,748), estimating the risk of newly diagnosed asthma and its associations with WTC work history, including mask or respirator use.

**Results:**

Newly diagnosed asthma was reported by 926 workers (3.6%). Earlier arrival and longer duration of work were significant risk factors, with independent dose responses (*p* < 0.001), as were exposure to the dust cloud and pile work. Among workers who arrived on 11 September, longer delays in the initial use of masks or respirators were associated with increased risk of asthma; adjusted odds ratios ranged from 1.63 [95% confidence interval (CI), 1.03–2.56) for 1 day of delay to 3.44 (95% CI, 1.43–8.25) for 16–40 weeks delay.

**Conclusions:**

The rate of self-reported newly diagnosed asthma was high in the study population and significantly associated with increased exposure to the WTC disaster site. Although we could not distinguish appropriate respiratory protection from inappropriate, we observed a moderate protective effect of mask or respirator use. The findings underscore the need for adequate and timely distribution of appropriate protective equipment and the enforcement of its use when other methods of controlling respiratory exposures are not feasible.

Following the attacks of 11 September 2001 (9/11) in New York City (NYC), an estimated 90,000 workers and volunteers were involved in rescue, recovery, clean-up, and support services (Dolan et al. 2006). The initial cloud of dust and smoke released during the collapse of the World Trade Center (WTC) towers consisted of pulverized building materials and products of combustion, which settled heavily over the WTC site over the first 12 hr (Landrigan et al. 2004). In the subsequent 2 weeks, resuspended particulate matter and fires were the predominant sources of airborne contaminants; smoldering fires continued to be a source of gaseous and particulate combustion products into December 2001 (Landrigan et al. 2004).

Studies have documented increased respiratory symptoms, severe persistent cough (“WTC cough”), reactive airways disease, and declines in pulmonary function among surviving first responders and other WTC workers after 9/11 [Banauch et al. 2006; Centers for Disease Control and Prevention (CDC) 2004; Feldman et al. 2004; Herbert et al. 2006; Herbstman et al. 2005; Levin et al. 2002; Prezant et al. 2002; Salzman et al. 2004; Skloot et al. 2004]. In each of these studies, declines in respiratory health were significantly associated with earlier time of arrival relative to the collapse of the towers. Likewise, being caught in the initial dust cloud on 11 September 2001 was significantly associated with increased respiratory symptoms among surviving occupants of damaged and destroyed buildings (Brackbill et al. 2006). Consistent with the results of these observational studies, mice that were experimentally exposed to high levels of fine particulate matter from the WTC site developed mild to moderate pulmonary inflammation and significant increases in airway hyperresponsiveness after acute exposure (Gavett et al. 2003).

Traditionally, the control of workers’ exposure to airborne contaminants involves a hierarchical approach that first aims to reduce or eliminate the source of pollution through engineering processes, such as by ventilation. Whenever effective engineering controls are not feasible, federal occupational safety standards require the establishment of a respirator program. The requirements include informing employees of respiratory hazards; selecting appropriate devices for routine use and foreseeable emergencies; providing respirator training, fit-testing, and medical evaluations; and conducting program evaluations [Occupational Safety and Health Administration (OSHA) 2006]. Underlying these regulations is the understanding that respirators should be used as a secondary means of controlling workers’ exposure to airborne contaminants, knowing that that no device is fully protective, and that the margin of safety afforded by their use is strongly dependent on selection, fit, and appropriate use (Martyny et al. 2002).

In the aftermath of the WTC disaster, engineering controls clearly were not feasible. Although steps were taken by a number of entities to provide respiratory protection to workers, adequate respiratory protection devices were not immediately or universally available or employed over the course of the rescue and recovery response. Self-contained breathing apparatuses typically used in firefighting are not designed for long-term use and generally were not employed at the site beyond the first day of the collapse (Feldman et al. 2004). The types of alternative devices reportedly worn by emergency responders and other workers ranged from surgical masks and ordinary nuisance dust masks, which lack certification for particulate exposure, to disposable N95 respirators and half- and full-face respirators with cartridges (Feldman et al. 2004; Prezant et al. 2002; Spadafora 2002). An inherent challenge was that many volunteers lacked prior experience and training in the use of personal protective equipment, including air-filtering respirators (Jackson et al. 2002). Fit checking and qualitative fit testing began shortly after 9/11, although it was inconsistently performed. Quantitative fit testing for respirators with cartridges began in late October (Lippy 2002). The percentage of workers using any respiratory protection increased over time (Feldman et al. 2004; Prezant et al. 2002), but overall consistency of use was generally low to moderate. Estimates of the number of frequent users in late September and October range from approximately 20–50% in observational data (Lippy 2002) to 50% (Banauch et al. 2006; Feldman et al. 2004) and 65% (Prezant et al. 2002) in self-reported data.

In this study we used data from the World Trade Center Health Registry (WTCHR) to describe the risk of self-reported asthma diagnosed by a health care provider after 9/11 and its association with timing and duration of work at the WTC site, as well as work-related risk factors for increased exposure to potential respiratory hazards. We also evaluated the use of masks or respirators of any type during work at the WTC site. We examined whether their use had a protective effect on the risk of newly diagnosed asthma, recognizing that not all devices provide equivalent protection against exposure to particulate matter and other air pollutants, and that improper fit and maintenance limit the amount of protection provided by any individual device.

## Methods

### Study population and exclusions

Workers and volunteers who conducted any rescue, recovery, clean-up, and/or volunteer tasks at the WTC Site, Staten Island, or in transport between these sites from 11 September 2001 to 30 June 2002 were recruited to enroll in the WTCHR, a collaborative effort of the NYC Department of Health and Mental Hygiene (DOHMH) and the U.S. Agency for Toxic Substances and Disease Registry, using lists of employees and volunteers involved in the response, where available, and via media and community outreach. The WTCHR protocol was approved by the institutional review boards of the CDC and the NYC DOHMH. Informed consent was obtained from all participants.

Of an estimated 91,469 workers involved in rescue, recovery, clean-up, and support services (Dolan et al. 2006), > 51,000 people were identified as potential registrants. Of these, 30,655 eligible participants completed an interview between 5 September 2003 and 20 November 2004, for an estimated coverage rate of 33.5%. A total of 29,626 registrants worked directly at the WTC site, defined as the area of Lower Manhattan west of Broadway, between Chambers Street to the north and Rector Place to the south. “The pile,” as it was commonly known, was further defined as the immediate area in the footprints of the collapsed buildings. The analyses presented in this article pertain to workers located in any area of the WTC site as defined above, distinguishing work on the pile.

We excluded workers who reported a diagnosis of asthma before 11 September 2001 (*n* = 2,773). An additional 129 registrants who were < 18 years of age at the time of the interview were removed from the sample to limit our case definition to adult-onset asthma. Registrants who were missing one or more of the primary analytic variables (sex, age, NYC residence status on 9/11, education, affiliated organization at the WTC site, smoking status, exposure to the initial dust cloud, first date of work, work history on the pile, use of masks and respirators, and asthma history) were also excluded (*n* = 976). Before these exclusions, when income was missing, we assigned registrants the median 2000 household income for their zip code (*n* = 2,601) (U.S. Census Bureau 2001a). Registrants missing work history information for a specific time period were excluded in analyses involving the relevant time period. The final full analytic sample consisted of 25,748 workers, of whom 9,171 (36.0%) were recruited using employee lists; the remainder contacted the registry after media and community outreach encouraged enrollment (self-identified).

### Questionnaire and data preparation

History of asthma was assessed using the question, “Have you ever been told by a doctor or other health professional that you had asthma?” If registrants responded positively, they were asked to further specify, “Did a doctor or other health professional first tell you that you had asthma before 9/11 or after 9/11?” We defined newly diagnosed asthma as cases diagnosed after 11 September 2001.

Workers’ affiliations at the WTC site were captured using 34 precoded categories and one open-ended “other” category. Open-ended responses were categorized and subjected to review by three raters. Disagreements between raters were reevaluated, and all final assignments were made by K.W. For the analyses, the resulting 52 organization types were grouped into seven overall categories based on assumed similarity of work tasks, although some overlap between categories was anticipated: *a*) fire and rescue, including the NYC Fire Department (FDNY) and other fire departments, task forces of the Federal Emergency Management Agency, and urban search and rescue teams; *b*) medical, including FDNY Emergency Medical Services (EMS) and other EMS teams, disaster medical and mortuary teams, medical examiner staff, and health care providers; *c*) law enforcement and military, including the NYC Police Department, Port Authority police, state and federal law enforcement, Coast Guard, National Guard, and all other armed forces; *d*) construction, including demolition, trucking, heavy engineering, utility work, environmental remediation and abatement, dust control; *e*) sanitation, specifically the NYC Department of Sanitation; *f* ) public agencies not already specified, including the Port Authority (nonpolice), the CDC, the U.S. Environmental Protection Agency, OSHA, the NYC DOHMH, and other city, state, and federal agencies; and *g*) volunteers and miscellaneous, including the Red Cross, Salvation Army, other volunteer agencies, unaffiliated volunteers, and all other non–disaster-related businesses and organizations.

Work history at the WTC site was documented across five analytic time periods aimed at representing a gradient of exposure to respiratory irritants from most intensive to least intensive: day 1 (11 September), day 2 (12 September), days 3–7 (13–17 September), weeks 2–15 (18 September–31 December 2001), and weeks 16–40 ( January–30 June 2002). Date of arrival and duration of work were coded using two open-ended questions in the baseline questionnaire specifying registrants’ first and last date of work, and three additional multiple-choice questions specifying the number of days registrants worked during 13 and 17 September and 18 September–31 December 2001 and 1 January–30 June 2002. The latter two time periods were recorded categorically. In summing duration of work, we used the midpoint of the indicated day range for these two time periods. For example, if the registrant selected “31–60 days,” we assigned 45.5 days of work for that time period. We also evaluated an alternate variable, which was the difference in days between the last and first date of work. We compared odds ratios (ORs) from logistic regression models for newly diagnosed asthma as predicted by quartiles of either variable, and the results were equivalent. We chose the summation method because it allowed for a larger sample size, due to workers missing exact start or end dates of work.

To document mask or respirator use, registrants were asked, “On [9/11, etc.] did you wear a mask all of the time, most of the time, some of the time, or not at all?” for each of the five time periods. The term “mask” was inclusive of disposable dust masks, surgical masks, disposable N95 particulate respirators, half-face and full-face respirators with particulate and/or chemical filtration cartridges, and self-contained breathing apparatuses. We estimated the number of days worked without a mask or respirator of any type by multiplying the number of work days by 0, 0.25, 0.5, and 1.0, corresponding to the four categories “all of the time,” “most of the time,” “some of the time,” and “not at all,” respectively. For example, a registrant who reported working 4 days during 13–17 September 2001 while using a mask or respirator “most of the time,” received a value of one unprotected work day (4 × 0.25 = 1) during that period. This variable was used in the arrival-stratified models described below. We also created a variable for initial mask or respirator use at the WTC site corresponding to one of the five analytic time periods used in the delayed-use model also described below.

Additional information collected on WTC work history included location on or off the pile in each of the five time periods. Pile workers were also asked to specify tasks they performed on the pile, including firefighting, attempted search and rescue, hand-digging, steel-cutting/torch operation, heavy equipment operation, and light construction. All registrants were asked if they were exposed to the dust cloud on 9/11. Smoking status at the time of the interview and demographic characteristics were also recorded, including sex, age, race, Hispanic ethnicity, education, and income, and residence on 11 September 2001.

### Statistical analyses

Descriptive statistics and multivariable analyses were conducted using SAS, version 9.1 (SAS Institute Inc., Cary, NC). We computed frequencies of demographic and work-related characteristics and described the 3-year risk of newly diagnosed asthma as well as its frequency across demographic and potential work-related risk factors. We tested for trends in arrival and duration of work using multiple logistic regression, where arrival period was modeled as an ordinal variable with values of 1–5, referring to the five ascribed time periods, and days of work were modeled continuously. We used multiple logistic regression to model newly diagnosed asthma as predicted by arrival date, duration of work, exposure to the dust cloud, work site organization, and work on the pile, controlling for age, sex, NYC residence, smoking status, and method of enrollment. Age was modeled continuously in all models using age + age^2^ terms to allow for a nonlinear relationship, because we observed that the youngest and oldest age groups had a lower rate of newly diagnosed asthma compared with the middle two age groups.

We then described the frequency of mask or respirator use by time period and affiliated organization at the site. We used multiple logistic regression to model newly diagnosed asthma as predicted by the number of days worked without any mask or respirator in each time period, stratified by arrival period. The arrival-stratified models likewise controlled for sex, age, NYC residence, and smoking status, as well as work days in the time period and total duration of work, exposure to the dust cloud, organization, work on the pile, and enrollment method.

Finally, we modeled the association between newly diagnosed asthma and delay between arrival and initial mask or respirator use, using a restricted subset of highly exposed workers who arrived on 11 September and worked in all subsequent time periods. For both the arrival-stratified models and the delayed-use model, we tested for potential interaction between working directly on the pile and working without a mask or respirator, and found that there was none. For comparability to other published data, we repeated the models in a restricted subset of firefighters. To evaluate potential self-selection bias, we again repeated the models and excluded all registrants who were self-identified rather than recruited from employee lists.

## Results

### Characteristics of the study population

WTC workers enrolled in the WTCHR were predominantly white, non-Hispanic males between 25 and 65 years of age (Table 1). Nearly half of the workers (47.8%) were residents of NYC on 11 September 2001. Compared with NYC residents, a larger proportion of WTC workers held at least a college degree (39.2% vs. 27.4%) or earned an annual household income > $35,000 (85.7% vs. 53.6%) (U.S. Census Bureau 2001b). The average prevalence of current smoking across the four age groups was lower than the NYC population average in 2004 for the same age groups (15.1% vs. 17.1%) (NYC DOHMH 2004).

The vast majority of workers arrived before 31 December 2001 (92.1%) (Table 1). Approximately one-third (34.2%) worked at the site for a week or less, another 31.1% worked as long as month, and the remaining 34.7% worked at the site for ≥ 31 days. Overall, 11,253 workers (43.7%) in the study population worked directly on the pile at some point during the disaster response.

The most common worksite affiliation among registrants was volunteer/miscellaneous (8,133; 31.6%). Police, law enforcement, and military agencies (4,906; 15.9%) and construction, utility, demolition, debris removal, and remediation unions and contractors (4,099; 15.9%) were the next largest groups, followed by firefighting and other rescue services (3,587; 13.9%), employees of other public agencies (3,216; 12.5%), the Department of Sanitation (1,603; 6.2%), and medical workers (1,014; 3.9%) (Table 1). Among registrants who worked on the pile, attempted search and rescue (71.5%) and hand-digging (71.5%) were the most frequently identified tasks. These were followed by firefighting (23.9%), light construction (21.7%), steel-cutting/torch operation (14.6%), and heavy equipment operation (11.5%).

### Newly diagnosed asthma and WTC work history

**Figure 1:**
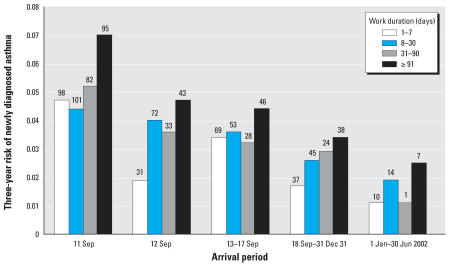
Three-year risk of newly diagnosed asthma in the study population by arrival period and duration of work at the WTC site. Numbers above bars indicate the number of cases.

We estimated an expected 0.3% 3-year risk of asthma, based on the reported incidence of asthma in the general adult population of 100/100,000 person-years (Reed 2006). A total of 926 registrants reported being told they had asthma for the first time after 9/11, which was equivalent to a 3-year risk of 3.6%; this was 12 times higher than expected. The frequency of workers reporting newly diagnosed asthma increased with arrival dates closer to the time of the collapse and with longer duration of work. The highest 3-year risk of newly diagnosed asthma was reported by workers who arrived on 11 September and worked > 90 days (7.0%) (Figure 1). When modeled simultaneously, the trends in earlier arrival (*p* < 0.001) and duration of work (*p* < 0.001) were independently significant. Furthermore, we observed a significant 2–3% increase in risk for every 10 days of work at the WTC site, controlling for arrival and exposure to the initial dust cloud (*p* < 0.001, data not shown).

After adjusting for demographic and work-related characteristics, the experience of being caught in the initial dust cloud on 9/11 remained a significant risk factor for newly diagnosed asthma, as did arrival during the first week (with ORs ranging from 1.81 for those who arrived on 11 September to 1.59 for those arriving later in the week), work duration > 90 days, and any history of working directly on the pile (Table 2). The 3-year risk of reported newly diagnosed asthma was elevated for all organization affiliations, ranging from 2.4 to 5.2%, compared with the expected background risk of 0.3%. In unadjusted models, the risk was significantly elevated for fire and rescue workers, medical workers, and police and military personnel compared with volunteers; however, organization type did not remain a significant predictor of newly diagnosed asthma in the adjusted model. Among workers who reported ever working on the pile, firefighting [OR = 1.61; 95% confidence interval (CI), 1.33–1.95], searching (OR = 1.71; 95% CI, 1.37–2.14), and hand digging (OR = 1.69; 95% CI, 1.35–2.12) were individually associated with an increased risk of asthma; however, in the fully adjusted model limited to pile-workers, the associations were not significant.

### Use of masks or respirators

**Figure 2:**
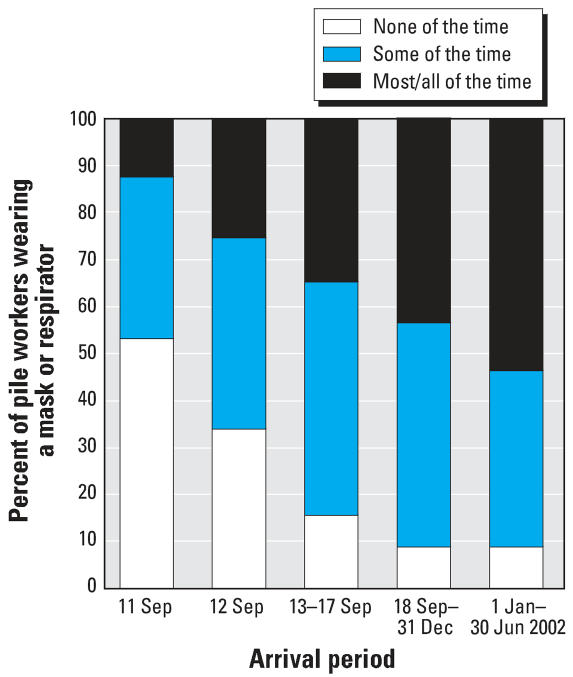
Frequency of reported use of mask or respirator by date among pile workers.

**Figure 3:**
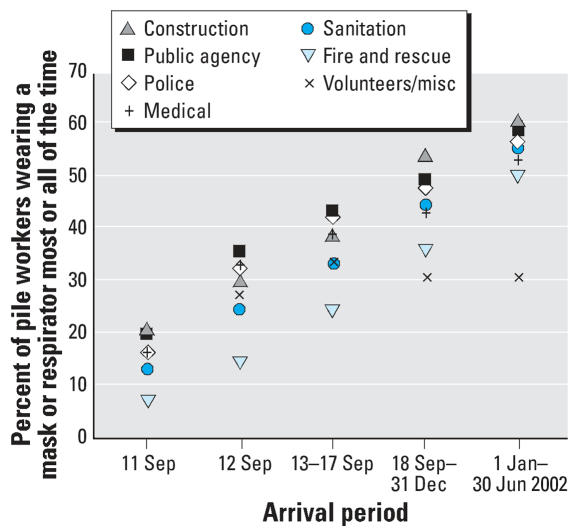
Percent of pile workers who reported wearing a mask or respirator most or all of the time, by organization.

In analyzing the frequency of reported mask or respirator use (Figures 2 and 3), we limited the study population in each time period to those who reported working on the pile, for comparability of exposure. The proportion of pile workers who reportedly wore a mask or respirator for at least some of the time increased from 50% on 11 September 2001, to > 80% after the first week (i.e., after 17 September 2001) (Figure 2). However, the percentage of pile workers who reported wearing a mask or respirator most or all of the time was smaller, peaking only near 50% after 31 December 2001. The variation between organization groups in this latter trend diminished by the final time period, with the exception of volunteers and miscellaneous workers (Figure 3).

### Assessment of days worked without a mask or respirator, by arrival time

We stratified the study population by time of arrival at the WTC site and evaluated the effect of days worked without a mask or respirator among workers arriving at the site for the first time during that period. Because of the small number of registrants who arrived in the final time period, 1 January–30 June 2002, and who reported newly diagnosed asthma (*n* = 32), regression models were limited to the first four arrival groups only. We did not restrict these analyses to pile workers, but we adjusted for pile work as described below.

Workers who arrived on 11 and 12 September were significantly more likely to report newly diagnosed asthma if they worked without any mask or respirator on either day [OR for 11 September, 1.51 (95% CI, 1.21–1.89); OR for 12 September, 1.42 (95% CI, 1.04–1.94)], controlling for work-related risk factors (exposure to dust cloud, affiliated organization, any pile work, and pile tasks), method of enrollment (self identified vs. recruited), demographic characteristics associated with newly diagnosed asthma, and smoking status (Table 3). A non-significant dose–response relationship was observed between newly diagnosed asthma and the number of days worked from 18 September to 31 December 2001 without a mask or respirator.

### Assessment of increasing delay in initial mask or respirator use

We also modeled the effect of incremental delays in the initial use of masks or respirators at the WTC site, restricting the analysis to workers with the greatest cumulative opportunity for exposure. These were workers who arrived on 11 September 2001 and worked in all subsequent time periods (*n* = 2,161).

Longer delays in the initial use of a mask or respirator were associated with significant increases in the risk of newly diagnosed asthma (Table 4). Compared to initiating use of a mask or respirator on 11 September, delays of 1 day (adjusted OR = 1.63; 95% CI, 1.03–2.56) and up to 1 week (adjusted OR = 1.62; 95% CI, 1.00–2.63) were associated with an approximately 60% increase in risk of newly diagnosed asthma. Further delays of up to 16 weeks and ≥ 16 weeks resulted in > 2-fold and 3-fold increases in risk, respectively.

### Subset analysis of firefighters

Because previously published studies of NYC firefighters did not detect a protective effect of masks and respirators (Banauch et al. 2006; Feldman et al. 2004; Prezant et al. 2002), we replicated our analyses with a subset of the study population restricted to the organization group comprised of firefighters and search and rescue teams. Again, we found a significant association between working without a mask or respirator on 11 September and newly diagnosed asthma (adjusted OR = 1.48; 95% CI, 1.02–2.15). When the delayed-use model was also restricted to fire and rescue workers only, the effects of delayed use were slightly larger than for the study population as a whole, and remained significant; delays of 1 day (adjusted OR = 2.24; 95% CI, 1.06–4.77) and up to 1 week (adjusted OR = 2.46; 95% CI, 1.22–4.96) were associated with > 2-fold increase in risk of newly diagnosed asthma, whereas delays of up to 16 weeks resulted in a > 3.5-fold increase in risk (adjusted OR = 3.70; 95% CI, 1.68–8.12), and delays after 1 January 2002 were associated with a nearly 5-fold increase in risk (adjusted OR = 4.78; 95% CI, 1.38–16.5).

### Assessment of potential self-selection bias

Self-identified workers had a significantly higher rate of newly diagnosed asthma (4.5%) compared with workers who were recruited via employee lists (2.0%) (OR 2.29; 95% CI, 1.94–2.69). We assessed the impact of potential selection bias by excluding all self-identified registrants from the arrival-stratified models. The detrimental effect of working without a mask or respirator on 11 September (adjusted OR = 1.87; 95% CI, 0.69–5.08) and 12 September (adjusted OR = 1.85; 95% CI, 1.02–3.34) was likewise evident and slightly increased in magnitude among list-recruited participants only; however, the CIs were wider, reflective of the loss of precision due to the reduced sample size. We did not repeat this analysis in the delayed-use model because the model required a restricted subset of the study population; a model further restricted to fire-fighters would have poor statistical power to assess associations.

## Discussion

Using data collected on the largest cohort of WTC rescue, recovery, clean-up, and volunteer workers, encompassing a diverse range of organizations involved at the site, we found that the risk of newly diagnosed asthma was 12-fold higher than the expected background 3-year risk in the general population (3.6% vs. 0.3%) (Reed 2006), and that there were significant increases in risk for earlier arrival, total duration of work, exposure to the dust cloud, and working on the pile at the WTC site. We also found that the timing of mask and respirator use was an important determinant of its protective effect, where earlier first-time use of masks and respirators at the site was significantly associated with decreased risk of newly diagnosed asthma.

The observed effect of arrival time in the study population was consistent with previous studies, which found that workers who arrived closer to the time of the collapse were more likely to experience respiratory symptoms and reduced pulmonary function after 9/11 (Banauch et al. 2006; CDC 2004; Feldman et al. 2004; Herbert et al. 2006; Herbstman et al. 2005; Levin et al. 2002; Prezant et al. 2002; Salzman et al. 2004; Skloot et al. 2004). Similar to results of Herbstman et al. (2005), we observed that total duration of work at the WTC site was also a significant risk factor for newly diagnosed asthma. We further demonstrated that the effect of working for an extended duration, especially > 90 days, was independent of workers’ arrival date and exposure to the initial dust cloud on 11 September 2001. Our results therefore suggest that the onset of asthma was not only associated with acute exposure to high levels of respiratory hazards but also with chronic exposure to presumably lower levels of airborne contaminants. Notably, Gavett et al. (2003) observed pulmonary inflammation and airway hyper-responsiveness in mice given a single, high-level exposure to WTC fine particulate matter; however, the study did not measure the effects of chronic low-level exposure.

The patterns of reported mask or respirator use in the present study were similar to those in previous studies based on self-reported use data (Banauch et al. 2006; Feldman et al. 2004; Prezant et al. 2002; Skloot et al. 2004). However, prior studies of surviving firefighters who worked at the WTC site did not detect a significant association between the use of masks and respirators and either reduced respiratory symptoms or changes in pulmonary function after 9/11 (Banauch et al. 2006; Feldman et al. 2004; Prezant et al. 2002). In the first two of these studies, use of any mask or respirator was summarized over the duration of the work period, both *a*) dichotomously, comparing frequent (protected) versus infrequent and nonusers (unprotected) (Prezant et al. 2002), and *b*) as a score indicator ranging from 0 (present at the site, unprotected) to 3 (not present at the site), which was averaged across four time periods (Feldman et al. 2004; Prezant et al. 2002). Given our finding that newly diagnosed asthma was significantly elevated among workers who had greater delays in initial use, we suggest that overall summary measures, such as those in the two aforementioned studies, might not capture the protective role of masks or respirators because they did not account for the timing of their use relative to workers’ arrival. A third study of firefighters compared frequent (protected) versus nonfrequent and nonuse of masks and respirators (unprotected) on the worker’s day of arrival (Banauch et al. 2006). Although this approach was similar to our models presented in Table 3, we quantified unprotected exposure as the estimated number of days worked without a mask or respirator during each arrival period, and stratified the regression model by arrival period.

In a study conducted among ironworkers, Skloot et al. (2004) compared workers who ever used a respirator (protected) to those who never used a respirator (unprotected), again as a summary measure over the duration of work at the site. The authors observed a significant protective association between the use of respirators with cartridges and changes in pulmonary function after 11 September 2001, but the association did not reach statistical significance for respiratory symptoms and was not significant in either case for dust masks alone. Neither the firefighters’ studies (Banauch et al. 2006; Feldman et al. 2004; Prezant et al. 2002) nor the ironworkers’ study (Skloot et al. 2004), however, measured the effect of increasing delay in mask or respirator use, which was unique to our study.

The rate of self-reported, newly diagnosed asthma in our study population was high; we estimated an expected count of 77 cases and observed 926. Although we hypothesized that firefighters, construction workers, and others would have higher background rates of adult-onset asthma than the general population, we found few data on the incidence of asthma across occupational groups and no published studies on the incidence of asthma in firefighters. One cohort study from Finland (Sauni et al. 2003) documented a 2% 5-year risk in construction workers. This was also elevated compared with the general adult population (0.4% vs. 0.1% 1-year risk), but was still lower than the estimated 1-year risk in the present study (1.2%).

Workers who developed asthma may have been more likely to enroll in the registry than workers who did not develop asthma. It is also possible that enrollees were more likely to misclassify their asthma status or time of diagnosis (before or after 11 September) than non-enrollees. For example, registrants experiencing a relapse of asthma may have selectively chosen “after 9/11” if they were unsure of an earlier diagnosis. We did not verify diagnoses using medical records and therefore cannot rule out overreporting by study participants. Health care provider behavior must also be considered, because WTC site workers may be more likely to be screened for respiratory illness than other workers and adults generally. Providers also may be more likely to offer a diagnosis of asthma in rescue, recovery, and clean-up workers. Notwithstanding, self-reported diagnosed asthma is a commonly used measure in the peer-reviewed literature and has been validated with very strong (99%) specificity in adults (Toren et al. 1993). Furthermore, we would not expect misclassification of disease to differ across categories of exposure intensity or duration, and we do not think it would have produced the exposure–response relationships we observed.

As an additional validation, we computed the prevalence of self-reported asthma diagnosed before 9/11 in the WTC worker population before excluding these cases (*n* = 2,773) from the study population. The prevalence was 9.8%, which was comparable with results for the U.S. adult population from the 2000 National Health Interview Survey (9.3%) and lower than the prevalence from the 2002 NYC Community Health Survey (12.0%) (Garg et al. 2003). It would not appear, therefore, that registrants as a whole were more likely to overreport asthma status. Finally, if we assumed at an extreme that none of the approximate 50,000–60,000 nonenrollees were diagnosed with asthma after 9/11, the 3-year risk of newly diagnosed asthma would be 1%, which is still > 3-fold higher than the background risk in the general adult population (0.3%).

As with any retrospective questionnaire, the results may also be subject to recall bias. It is possible that workers who developed asthma might have underreported mask use in an attempt to explain their disease. It is equally possible that workers overreported mask use to avoid blame for noncompliance. The net direction of the resulting bias is unknowable, although it is unlikely to act in such a way as to produce an apparent trend between newly diagnosed asthma and increasing delay in mask or respirator use.

There was potential misclassification in the estimation of time worked at the WTC site because of differing work shift lengths. Our analyses assumed 1 day’s work was equivalent across the study population, whereas shift length may have varied between occupational groups. As a result, the number of days worked without masks or respirators would be misclassified, with the highest exposure group tending to be combined with less-exposed groups. Such error would most likely bias the results toward the null. Of note, we observed an increase in the magnitude of the association between newly diagnosed asthma and working without masks and respirators in the models restricted to firefighters, who, anecdotally, have been reported to have routinely worked long shift lengths.

Although we found that mask or respirator users were more likely to have worked on the pile (data not shown), a significant risk factor for newly diagnosed asthma that we controlled for in our models, it is possible that mask use was also associated with protective behaviors such as working shorter shift lengths (not measured) and not smoking. We controlled for smoking status in our models, even though we did not detect evidence of confounding in this study. Again, we do not suspect that confounding by an unmeasured protective factor would otherwise explain the observed trend between delay in mask use and risk of newly diagnosed asthma. It is possible, however, that the onset of respiratory symptoms may have prompted workers to begin using a mask or respirator, in which case the results would be biased toward the null.

A central limitation of this study was the inability to distinguish the type of mask or respirator used, which was not assessed in the questionnaire. In addition, the baseline questionnaire did not assess previous training in the use of respiratory protection equipment, degree of fit-checking, fit-testing, or maintenance of respirators used at the site. Were we able to measure and control for these variables in the analyses, we would expect the magnitude of the effect of appropriate respiratory protection to be in fact greater than that which we observed. The first follow-up survey of registrants, conducted in 2007, includes questions on type(s) of masks or respirators worn, training, access to fit-testing, qualitative fit-checking, and respirator maintenance.

A number of recommendations were voiced by participants at a national meeting conducted in December 2001 that was attended by emergency responders, law enforcement, construction and trade workers, health and safety workers, and local and federal agency workers involved in the responses to events of 9/11, the Oklahoma City bombing, and the 2001 anthrax incidents. Participants suggested a need for planning to ensure the rapid supply of appropriate respiratory and other personal protective equipment for workers who may be called to respond to disasters. Our findings in fact demonstrate the benefit of the rapid initiation of respiratory protection use. Other recommendations concerned the need for anticipatory preevent and early on-site training in the use of different types of masks and respirators; increased on-site risk communication regarding respiratory hazards; and planned, independent regulatory oversight of respiratory protection programs and other areas of occupational safety and health via incident command structures for disaster response (Jackson et al. 2002).

## Conclusion

The use of masks and respirators at the WTC site did not eliminate the risk of newly diagnosed asthma in the study population; however, we did observe evidence of a protective effect, even given the limitations already documented. It is reasonable to conclude that the early initiation and consistent use of appropriate respiratory protection may have further prevented additional cases of new-onset asthma. As such, the findings underscore the importance of *a*) preparedness for the health and safety of workers who may be called to respond to a disaster through anticipatory training, *b*) the adequate and timely distribution of appropriate personal protective equipment, and *c*) the enforcement of respiratory protection programs when other methods of controlling exposure to hazardous airborne contaminants are not feasible.

## Correction

In the manuscript originally published online, the number of eligible participants (32,705) was incorrect; it has been corrected here to 30,655.

## Figures and Tables

**Table 1:** Number (%) of workers in the study population (*n* = 25,748) by selected demographic characteristics, smoking status, and WTC work history.

Variable	No. (%)
Sex	
Male	20,394 (79.2)
Female	5,354 (20.8)
Age on 11 Sep 2001 (years)	
18 to < 25	1,346 (5.2)
25 to < 45	15,599 (60.6)
45 to < 65	8,319 (32.3)
≥65	484 (1.9)
Race	
White, non-Hispanic	18,670 (72.5)
Black, non-Hispanic	2,246 (8.7)
Hispanic or Latino	3,464 (13.5)
Asian	607 (2.4)
Multiple	457 (1.8)
Other or unknown	304 (1.2)
Income (US$)	
< 35,000	3,673 (14.3)
35,000 to < 100,000	16,286 (63.3)
≥100,000	5,789 (22.5)
Education	
Did not complete high school	1,292 (5.1)
High school graduate or GED	6,331 (24.6)
Some college	8,045 (31.3)
College or post-graduate degree	10,080 (39.2)
Residence on 11 Sep 2001	
NYC	12,282 (47.8)
Outside NYC	13,466 (52.3)
Smoking status on interview date	
Current smoker	4,434 (17.2)
Former smoker	6,930 (26.9)
Never smoked	14,384 (55.9)
Date of arrival at the WTC site	
11 Sep 2001	7,339 (28.5)
12 Sep 2001	5,204 (20.2)
13–17 Sep 2001	5,398 (21.0)
18 Sep–31 Dec 2001	5,807 (22.6)
1 Jan–30 Jun 2002	2,000 (7.8)
Duration of work at the WTC site (days)	
1–7	8,754 (34.0)
8–30	8,002 (31.1)
31–90	4,279 (16.6)
> 90	4,713 (18.3)

**Table 2:** Unadjusted and adjusted ORs for newly diagnosed asthma, predicted by work history characteristics (*n* = 25,748).^a^

WTC work-related factors	No.of cases (%)	Unadjusted OR (95% CI)	Adjusted OR (95% CI)
Arrival date			
11 Sep 2001	376 (5.1)	3.32 (2.31–4.78)	1.81 (1.19–2.74)
12 Sep 2001	179 (3.4)	2.19 (1.50–3.20)	1.55 (1.03–2.33)
13–17 Sep 2001	195 (3.6)	2.30 (1.58–3.36)	1.69 (1.13–2.51)
18 Sep–31 Dec 2001	144 (2.5)	1.56 (1.06–2.31)	1.25 (0.84–1.86)
1 Jan–30 Jun 2002	32 (1.6)	Reference	Reference
Duration of work at WTC site (days)			
> 90	229 (4.9)	1.78 (1.48–2.14)	1.74 (1.43–2.12)
31–90	168 (3.9)	1.43 (1.17–1.74)	1.18 (0.95–1.45)
8–30	285 (3.6)	1.29 (1.09–1.53)	1.20 (1.00–1.44)
1–7	244 (2.8)	Reference	Reference
Organization			
Fire and rescue	188 (5.2)	1.88 (1.55–2.31)	1.20 (0.93–1.54)
EMS and medical, medical examiner	49 (4.8)	1.73 (1.26–2.37)	1.11 (0.79–1.54)
Law enforcement and military	190 (4.6)	1.66 (1.36–2.01)	0.99 (0.79–1.25)
Construction, utilities, remediation	118 (2.9)	1.01 (0.81–1.26)	0.98 (0.77–1.26)
Sanitation	38 (2.4)	0.83 (0.58–1.17)	1.05 (0.72–1.53)
Public agency, not already specified	111 (3.5)	1.22 (0.97–1.53)	0.92 (0.72–1.17)
Volunteers and miscellaneous	232 (2.9)	Reference	Reference
Exposed to 9/11 dust cloud			
Yes	476 (4.9)	1.77 (1.55–2.01)	1.28 (1.09–1.50)
No	450 (2.8)	Reference	Reference
Any work on the pile			
Ever	505 (4.5)	1.57 (1.38–1.79)	1.30 (1.11–1.53)
Never	421 (2.9)	Reference	Reference

a The adjusted model controls for female sex, age, age^2^, NYC residence on 11 September 2001, affiliated organization, duration of work, exposure to the dust cloud, and work on the pile.

**Table 3:** Unadjusted and adjusted ORs for newly diagnosed asthma, predicted by number of days worked at the WTC site without masks and respirators in each time period, among workers who arrived during that time.

				Newly diagnosed asthma	
Arrival date	No. of days worked without mask or respirator during time period	Total no. of workers in time period^a^	Percent reporting newly diagnosed asthma	Unadjusted OR (95%CI)	Adjusted OR (95% CI)^b^
11 Sep 2001	0	3,620	4.0	Reference	Reference
	1	3,683	6.3	1.64 (1.32–2.02)	1.51 (1.21–1.89)
12 Sep 2001	0	3,377	2.9	Reference	Reference
	1	1,774	4.5	1.54 (1.14–2.09)	1.42 (1.04–1.94)
13–17 Sep 2001	0	1,683	3.3	Reference	Reference
	1–4	3,066	3.8	1.16 (0.84–1.61)	1.13 (0.80–1.59)
	5	227	3.5	1.06 (0.50–2.26)	1.20 (0.54–2.67)
18 Sep–31 Dec 2002	0	783	2.0	Reference	Reference
	1–7	2,175	2.0	0.97 (0.54–1.73)	1.21 (0.66–2.23)
	8–30	2,102	2.7	1.34 (0.76–2.34)	1.08 (0.55–2.13)
	≥31	619	4.0	2.02 (1.07–3.81)	1.61 (0.69–3.78)

a Excludes workers’ missing responses specific to time period of analysis: 11 Sep (36; < 1.0%); 12 Sep (53; < 1.0%); 13–17 Sep (424; 7.8%); 18 Sep–31 Dec (128; 2.2%).

b Adjusted models control for female sex, age, age^2^, NYC residence on 11 September 2001, smoking status, affiliated organization, duration of work, exposure to the dust cloud, pile work, and pile tasks.

**Table 4:** Unadjusted and adjusted ORs for newly diagnosed asthma predicted by increasing delays in the use of masks and respirators among workers who arrived on 11 September 2001 and worked in all time periods (*n* = 2,037).

				Newly diagnosed asthma	
Initial use of masks and respirators	Amount of delay	No. of workers	Percent reporting newly diagnosed asthma	Unadjusted OR (95% CI)	Adjusted OR (95% CI)^a^
11 Sep 2001	0 days	1,022	4.9	Reference	Reference
12 Sep 2001	1 day	483	8.1	1.77 (1.13–2.76)	1.63 (1.03–2.56)
13–17 Sep 2001	1 day to < 1 week	399	8.8	1.98 (1.25–3.13)	1.62 (1.00–2.63)
18 Sep–31 Dec 31 2001	1 week to < 16 weeks	171	10.5	2.27 (1.27–4.07)	2.28 (1.22–4.25)
1 Jan–30 Jun 2002	16 weeks to 40 weeks	35	14.3	3.39 (1.25–9.15)	3.46 (1.22–9.81)
Never	—	51	15.7	3.93 (1.74–8.87)	3.44 (1.43–8.25)

a Adjusted model controls for female sex, age, age^2^, NYC residence on 11 September 2001, smoking status, affiliated organization, duration of work, exposure to the dust cloud, pile work, and pile tasks.
